# Inoculation density and nutrient level determine the formation of mushroom-shaped structures in *Pseudomonas aeruginosa* biofilms

**DOI:** 10.1038/srep32097

**Published:** 2016-09-09

**Authors:** Azadeh Ghanbari, Jaber Dehghany, Timo Schwebs, Mathias Müsken, Susanne Häussler, Michael Meyer-Hermann

**Affiliations:** 1 Department of Systems Immunology and Braunschweig Integrated Centre of Systems Biology, Helmholtz Centre for Infection Research, Braunschweig, Germany; 2 Institute for Molecular Bacteriology, Twincore, Centre for Experimental and Clinical Infection Research, Hannover, Germany; 3 Department of Molecular Bacteriology, Helmholtz Centre for Infection Research, Braunschweig, Germany; 4 Institute for Biochemistry, Biotechnology and Bioinformatics, Technische Universität Braunschweig, Braunschweig, Germany

## Abstract

*Pseudomonas aeruginosa* often colonises immunocompromised patients and the lungs of cystic fibrosis patients. It exhibits resistance to many antibiotics by forming biofilms, which makes it hard to eliminate. *P*. *aeruginosa* biofilms form mushroom-shaped structures under certain circumstances. Bacterial motility and the environment affect the eventual mushroom morphology. This study provides an agent-based model for the bacterial dynamics and interactions influencing bacterial biofilm shape. Cell motility in the model relies on recently published experimental data. Our simulations show colony formation by immotile cells. Motile cells escape from a single colony by nutrient chemotaxis and hence no mushroom shape develops. A high number density of non-motile colonies leads to migration of motile cells onto the top of the colonies and formation of mushroom-shaped structures. This model proposes that the formation of mushroom-shaped structures can be predicted by parameters at the time of bacteria inoculation. Depending on nutrient levels and the initial number density of stalks, mushroom-shaped structures only form in a restricted regime. This opens the possibility of early manipulation of spatial pattern formation in bacterial colonies, using environmental factors.

Biofilms are communities of bacterial cells which are associated with an interface and surrounded by an extracellular matrix^1^,^2^. They cause a wide range of infections^3^,^4^, contaminate water systems^5^,^6^ and cause biofouling of industrial equipment^7^,^8^. Moreover, biofilms are notoriously resistant to antibiotics: biofilm bacteria can be more resistant to antimicrobial stress than their free-swimming counterparts^4^,^9^,^10^,^11^.

Biofilm formation is often depicted as a series of different steps^12^, starting with surface attachment of planktonic cells. When some coloniser cells adhere to the surface, the biofilm grows through a combination of cell division and recruitment (of planktonic cells). This leads to formation of microcolonies. When the initial microcolonies are established, biofilms undergo a maturation step and form larger structures. Surface-associated cell motility is critical for the development of *P*. *aeruginosa* biofilms and mushroom formation. Confocal laser scanning microscopy (CLSM) shows that an immotile subgroup of *P*. *aeruginosa* cells forms microcolonies on the surface by clonal growth^13^. These microcolonies serve as the lower parts of the mushroom-shaped structures and are often called *stalks*. A motile subgroup of *P*. *aeruginosa* cells colonises the top of these stalks^13^ and forms the mushroom cap. This shows that cap formation is an active process formed by cell aggregation, in contrast to cell growth and division. The differentiation of *P*. *aeruginosa* cells into motile and immotile subpopulations is affected by the environment^14^.

Dispersion or the spread of bacterial cells involves detachment of a subpopulation of biofilm cells, which retain flagellum-mediated motility (swimming) and escape from the biofilm. Counter-intuitively, this detachment does not necessarily happen at the biofilm surface. In mature biofilms, some bacterial cells start rapid flagellum-driven movements inside the compact microcolonies^15^,^16^. Loosening of the microcolonies eventually leads to the escape of the planktonic cells which leave cavities behind in the biofilm centre^17^. The escaped planktonic cells presumably form new colonies elsewhere with better access to nutrients.

Several mathematical models have been developed to study bacterial colonies and biofilms from different aspects. These topics range from population dynamics^18^,^19^ and morphological development^20^,^21^,^22^,^23^,^24^,^25^ to the evolutionary interactions of bacterial species^26^,^27^,^28^,^29^, as well as treatment of bacterial biofilms^30^. The mathematical approaches in use include continuum models^22^,^25^,^26^,^30^,^31^ as well as discrete (cell-based) models in two dimensions (2D)^19^,^20^,^21^,^28^,^29^,^32^ or three dimensions (3D)^18^,^23^,^24^.

Picioreanu *et al*. developed a 3D agent-based model to study the surface roughness and porosity of a single-species biofilm under different growth conditions^20^. They found that porous biofilms form in “substrate-transport-limited” regimes. Compact biofilms form when the growth rate is limited. Nadell *et al*. further investigated the mixing state of cell lineages within a single-species bacterial colony^28^. Using a 2D agent-based model, they showed that reducing the substrate availability affects the morphology of the bacterial colony by shifting it from a well-mixed to a segregated lineage regime. Some other models have investigated the relations between the high-order bacterial patterns (morphology) and the evolution of bacterial species therein^26^,^27^,^28^,^29^. A 2D agent-based model was used to show that the emergence of biofilm patterns and evolutionary competition among its bacterial cells are linked: both are driven by competition for nutrients and the mechanical interactions of bacterial cells^26^. A similar model was used to investigate the evolution of bacteriocin production in biofilms^29^. It was shown that the biodiversity of bacterial strains is maintained in highly segregated biofilms. However, it rapidly decreases in well-mixed biofilms and becomes sensitive to the critical bacteriocin range^29^.

The surface motility of bacterial cells was first considered in an agent-based model^24^. This model reproduced the experimentally observed^33^ tendency of the motile cells to form flat biofilms, unlike immotile cells which form round colonies by clonal growth. However, it proposed detachment and reattachment of the motile cells as a possible mechanism leading to cap formation (by motile cells) on top of the round colonies (built by immotile cells)^24^. Using a continuum model, Miller *et al*.^25^ investigated mushroom formation in *P*. *aeruginosa* biofilms. They assumed an accelerated expansion of the cap-forming bacterial subpopulation relative to the stalk-forming subpopulation, driven by production of extracellular polymeric substances, which, in this model, was assumed to drive the bacteria apart faster than they grow. The model successfully produced the mushroom-shaped structures. The mushroom caps in this model developed from the top of the colonies^25^. This is analogous to the previous models^22^,^31^, which stated that the growing biofilm fingers, induced by nutrient limitation^20^,^21^, eventually become mushrooms. Although this scenario might happen in some biofilms, experiments show different dynamics for mushroom formation in *P*. *aeruginosa* biofilms. The direct visualisation of *P*. *aeruginosa* cells via time-lapse CLSM shows that the motile bacterial subpopulation actively migrates onto the existing microcolonies (stalks) and forms caps^13^. Later, the biomass of these caps may increase to some extent due to growth. However, according to the same study, the immotile cells do not grow significantly during the period of migration of motile cells on top of the stalks^13^.

Previously published models have mostly focused on biofilm development via passive motion of the cells. Bacterial motility models have used active cell motility to explain the physical aspects of the collective motion of cells^34^ rather than their ultimate effects on biofilm morphology. The current model investigates the influence of active cell motion and the nutrient level on biofilm morphology. A novel feature of the current model, which includes motile and immotile cells, is that the motile cells can move not only on the substratum but also on the surface of a 3D biofilm (see Cell motility for details). The inclusion of both cell types allows us to interpret conflicting quantitative measurements at the single-cell level: On one hand, collective cell motion onto the stalks has been observed^13^,^35^,^36^. This speaks against mushroom formation by cells growing on the top of the stalks, which, on the other hand, is supported by the observation that nutrient gradients are directed away from pre-existing microcolonies^15^,^19^,^37^,^38^. The model resolves this contradiction by showing that a critical density of stalks is required to allow for collective cell motion onto the stalks.

## Model Description

The agent-based spatial model consists of two main parts: (A) the dynamics of bacteria, the agents; (B) diffusion of a soluble substrate, described by the reaction–diffusion equations (see Substrate diffusion). The model parameters are summarized in Supplementary Information.

### Simulation box

A Cartesian simulation box of dimensions *l*_*x*_ × *l*_*y*_ × *l*_*z*_ is considered. All simulations begin with inoculations at random positions on the surface of the impenetrable bottom (*z* = 0) of the simulation box. Periodic boundary conditions are applied in the *x* and *y* directions. For the boundary conditions of the soluble substrates, please see Substrate diffusion. Bacteria can displace continuously in the space. *P*. *aeruginosa* cells are approximated as spherical objects. A *P*. *aeruginosa* cell is assumed to have a radius *R*_0_ = 0.68 *μm*, a volume of *V* = 1.33 *μm*^3^ and a mass of *M*_0_ = 384 *fg*^32^.

### Cell–cell and cell–substratum interactions

To define and fine-tune the physical interactions of the model cells with each other, and also with the substratum, we need to define their interaction potentials. Intercellular interactions are described by a Lennard–Jones (LJ) potential. As the cell radii are dynamic quantities, the LJ potential is defined as a function of cell overlap, not of the distance between cell centres. The attractive part of the LJ potential represents cell–cell adhesion, whereas the repulsive part prevents unrealistic interpenetration of cells. The LJ potential between cells and the substratum is assumed to be two orders of magnitude stronger than the cell–cell LJ potential, to represent an impenetrable surface. During cell division, the LJ potential is replaced by a spring potential in order to avoid artefacts (see Cell growth and division). The net force 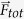 experienced by each cell is the sum of all interactions with neighbouring cells 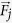 and the substratum 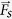.

### Simulation time step

The whole simulation time is discretised with a time step *δt*. At every time step, the new state of each cell (e.g. position and direction) and the local concentration of solutes throughout the simulation box are updated. The time step is dynamically adapted during simulations, so that no cell moves more than its average cell size (2*R*_0_) per time step or artificially passes through a different cell. The time step *δt*_*i*_ at the *i*^th^ step is determined by the largest net force experienced by a cell. *δt* is further limited by the condition that no cell can grow more than 0.1 × *M*_0_ per time step. In practice, this criterion is only met at the initial phases of biofilm growth at very high nutrient concentrations when cells are not close enough to interact.

### Cell motility

The model distinguishes between passive and active cell motility. Cells that are only passively displaced because of cell growth and division are called *immotile*. During cell growth, the radius and mass of the cells increase (see Cell growth and division) and induce a repulsive force at a threshold overlap in the LJ potential (see Cell–cell and cell–substratum interactions). The same passive displacement may happen during division. This passive displacement of immotile cells is also called *shoving* in the literature. In addition to passive displacement, cells may actively move on the substratum or the biofilm surface. These cells are called *motile* cells. The direction of movement is determined by chemotaxis (see Chemotaxis) and remains unchanged until it has moved a *persistence length*^39^.

**Figure 1:**
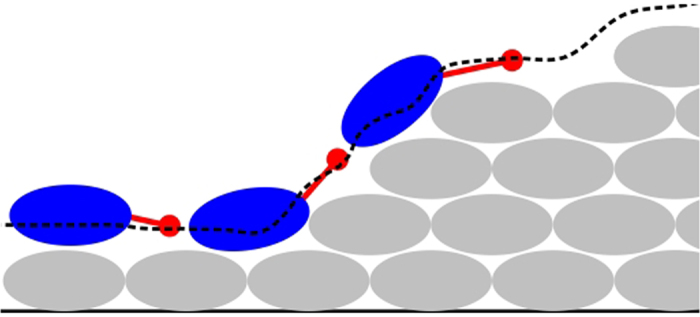
Schematic view of cell motility. Motile cells (blue) move on the biofilm (grey cells) surface as well as on the dish bottom. To perform cell motion on the biofilm surface, a superficial mesh (dashed black line) is constructed at each time step. A motile cell is pushed in the direction shown by the red line.

*P*. *aeruginosa* cells actively spread on a surface by flagellum-mediated swarming and pilus-mediated twitching motilities^40^,^41^. Cell movement and reorientation are the basis of chemotaxis. In *P*. *aeruginosa* cells, chemotactic cell movement mainly involves flagella^35^,^40^, although there is also evidence for the role of twitching motility^42^,^43^. In the present model, we assume a simple chemotactic cell motion that does not involve explicit pilus or flagellum dynamics. Figure 1 schematically shows a motile cell (blue) moving on the surface of a cell colony (grey). The biofilm surface is approximated by a dashed line (black). While the *P*. *aeruginosa* cell is moving on the surface, it passively follows the eventual topology of the surface. In the model, the biofilm surface (corresponding to the black dashed line in Fig. 1) is determined at every time step. The biofilm surface is represented by a cubic mesh of discrete points with a mesh size of *h* = *R*_0_/2. The choice of *h* < *R*_0_ makes the surface mesh finer than the cell size. The motile cell chooses an anchor point on the surface mesh according to its direction of migration. The anchor points are represented by red circles in Fig. 1. The motile cell is then pushed by 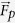 in direction of the anchor point for one time step. We have not assumed any active cell detachment mechanism in the model but instead assume that detaching cells, in reality, leave the biofilm community by swimming away.

The total force 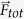 on the cell is determined by:


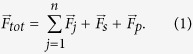


Cell displacement is determined by solving the over-damped equations of motion:


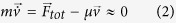


where 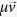 denotes the isotropic friction of a sphere of radius *R* in a medium of viscosity *η*^44^: *μ* = 6*πηR*. No rotational degree of freedom or cell Brownian motion is considered. Using these approximations, the cell speed is given by 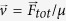 at each time step.

### Chemotaxis

*In silico*, motile cells follow chemical gradients, such as nutrients. Migration of motile cells is characterised by the direction of movement, the speed, and by a persistence time. When a motile cell has moved at least one persistence time in one direction, a new direction of movement is attributed to the cell. The choice of a new direction depends on the local level of a given signal. To follow the gradient of the signal, the cell virtually moves in *n*_*d*_ = 10 random directions^45^ and picks the direction with the highest signal level. When the signal at all *n*_*d*_ virtual positions is above a threshold of *c*_*u*_, the directional persistence of the cell is halved, effectively reducing the distance achieved. Conversely, when the signal is below a critical level *c*_*l*_, the cell chooses a random direction and follows it for one persistence time. This mimics an active search for more signals at levels that cannot be distinguished by bacteria^46^,^47^.

Two chemotactic signal-induced motions are assumed in the system. The first one is nutrient chemotaxis, by which motile cells follow the substrate gradient. This mechanism represents competition for nutrients among cells. The second one is an attractant signal-based motion by which motile cells are attracted to the stalks. One example of such a signal could be the quorum sensing-mediated interaction of bacterial subpopulations^48^. An attractant signal is assumed to be constantly secreted by the immotile cells only, at a rate that is half of the cell maintenance rate: *m*_*q*_ = *m*/2 (see Cell growth and division). The attractant signal is assumed to diffuse with the same diffusion coefficient as the substrate. We further assume that the attractant signal level reaches zero at the top of the simulation box (*z* = *l*_*z*_) by imposing a Dirichlet boundary condition (see Substrate diffusion). These two signals are implemented in the model in order to investigate whether and how attraction or competition mechanisms contribute to mushroom formation. Cells determine the local concentration of signals at every time step (see Substrate diffusion).

### Cell growth and division

In the model, the rate of cell growth and therefore the frequency of cell division depend on the local micro-environmental conditions. A cell stays in the growth phase until its mass meets a cell division criterion. The time-length of this growth phase depends on nutrient availability. Upon division, the cell is replaced by two daughter cells of the same type (motile or immotile). The division phase is assumed to last 30 min. If the local substrate level falls below a threshold during the growth phase, the cell enters a paused state and re-enters the growth phase when the substrate level is above the threshold again. We assumed Monod kinetics for the substrate uptake rate of cells, following Kreft *et al*.^32^. Cells divide when their mass reaches a value of 2*M*_0_. The total mass of the two daughter cells is equal to that of the mother cell, *M*_*m*_ (mass conservation). Their mass is randomly chosen between 40% and 60% of *M*_*m*_. This simple assumption removes any artificial synchronisation of cell division.

The initial direction of division is chosen randomly from a uniform distribution. The effective division direction may change due to interactions with the surrounding cells. To avoid a sudden overlap with the surrounding cells at the beginning of division, the daughter cells are placed at a distance 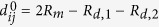, where *R*_*m*_ and *R_d_* are the radii of the mother and daughter cells, respectively. The maximum distance spanned by the daughter cells (in the centre-centre direction) is then equal to the diameter of the mother cell. To represent the separation force between the daughter cells, their interaction potential is replaced by a spring potential. The equilibrium length of the spring grows linearly at each time step from 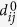 at the beginning to 
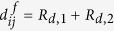
 at the end of division. The spring potential between the daughter cells is typically 100-fold stronger than the LJ potential between a daughter cell and its neighbours. This restoring force does not lead to harmonic oscillations because of the overdamped approximation (see Cell–cell and cell–substratum interactions). This ensures that the contact between the daughter cells will not be lost through interactions with surrounding cells.

### Substrate diffusion

We assume that the substrate reaches the biofilm mainly by diffusion near biofilm surface^37^ and passively diffuses into the biofilm. Therefore, to represent the transport of the substrate, we solve a reaction–diffusion equation in the simulation box. The processes of diffusion for both signals (see Chemotaxis) are handled similarly, as follows. For simplicity, we explain the details only for nutrients. The diffusion process is described by the reaction–diffusion equations in the model below:


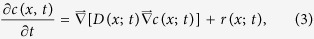


where *c*(*x*, *t*) denotes the local substrate concentration, *D*(*x*; *t*) is the local diffusion coefficient and *r*(*x*; *t*) is the substrate reaction rate. This equation is solved in the steady state 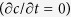. Equation (3) is discretised on a cubic lattice, with a lattice constant *l*_*d*_ = 2 *μm* that is slightly larger than the average cell diameter^44^. At every time step, the local substrate concentration, *c*(*x*, *t*), is determined using a trilinear interpolation from the concentrations at the eight closest lattice nodes^44^. Similarly, the reaction rates created by the cells (Cell growth and division) are distributed on the closest lattice nodes. The value of the effective diffusion coefficient in a biofilm (*D*_*e*_) is usually less than that in water *D*_*aq*_. This is because of the presence of bacterial cells, the extracellular matrix and abiotic substances inside the biofilm^37^. The typical value of *D*_*e*_/*D*_*aq*_ in biofilms is 0.6 for light gases (such as oxygen and carbon dioxide) and 0.25 for most organic compounds^37^. We assume *D*_*e*_/*D*_*aq*_ = 0.25. The local diffusion coefficients are updated at every time step based on the current cellular positions.

At the bottom of the diffusion lattice (substratum), no-flux von Neumann boundary conditions are assumed: 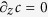. To represent the bulk liquid at the top of the simulation box, Dirichlet boundary conditions are applied: 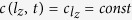. In our reference model 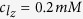 is assumed, unless otherwise indicated. This boundary condition reflects the flow chamber situation with a constant replenishment of nutrients at the top of the chamber. In the *x* and *y* directions of the diffusion lattice, periodic boundary conditions are assumed. The initial condition 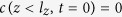 is used.

### Experimental methods

Biofilms under flow conditions were cultivated and visualised as previously described with slight modifications^49^, as follows. In brief, different inocula with optical densities (OD600) of 0.0001, 0.005 and 0.05 of PAO1 wild-type cells constitutively expressing green fluorescent protein (GFP) were injected into a six-channel μ-Slide (Ibidi, Germany). After 30 min of bacterial settlement, the flow was started and kept constant throughout the experiment to supply the biofilms with fresh FAB medium (AB minimal medium with 10 μM Fe-EDTA replacing FeCl_3_) containing 0.3 mM citrate as a carbon source and the dye propidium iodide (PI) to stain dead cells. CLSM was performed with an inverted Leica SP8 system after 72 h at various positions (distance between positions = ~1 mm) in the different channels. GFP signals were detected using a multi-argon laser (excitation wavelength = 488 nm) and an emission range of 500–550 nm and PI signals with a 561 nm laser and a range of 675–750 nm. Image stacks were acquired for a total height of 100 μm with a *z*-step size of 3 μm. 3D reconstruction and calculation of image objects were performed with the software Imaris 7.6, using the same settings for all image stacks. Stacks were thresholded and objects were determined, including a splitting option to separate single events (see *Supplementary Information* for further details).

## Results and Discussion

**Figure 2:**
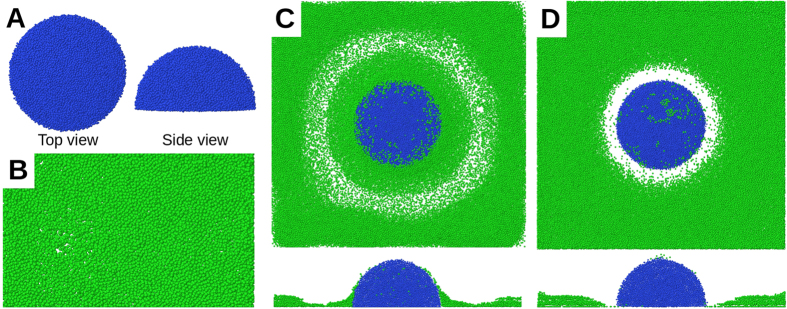
Different colonies formed by motile and immotile cell types. (**A**) Top and side views of a simulated colony of immotile cells only, grown on the substratum. (**B**) Top view of a simulated colony of motile cells only, grown on the substratum. (**C**) Cooperation: the motile cells surround the immotile cell stalk because they are attracted by an attractant signal. (**D**) Competition: In the absence of an attractant signal, nutrient chemotaxis repels the motile cell from the single stalk along the nutrient gradient. Blue and green colours represent the immotile and motile cells, respectively.

When immotile cells are placed on the model substratum, colonies of immotile cells form; these are called *stalks* in this text. The morphology of a stalk tends towards a hemisphere after long periods (Fig. 2A). This is due to the stochastic nature of the division direction, which forces initial differences to average out in the *x* and *y* directions after some time. The dimension of a stalk is limited by nutrient availability and the effective diffusion coefficient inside the colony^37^. Inoculation with motile cells only leads to formation of a flat colony, which, after a long time, uniformly covers the whole substratum (Fig. 2B). Therefore, having only motile cells in the model is insufficient to form mushroom-shaped structures. In the following, an equal number of motile and immotile cells will be inoculated at the same time. Besides physical cell–cell interactions (Cell–cell and cell–substratum interactions), motile and immotile cells can indirectly interact. They may compete via nutrient chemotaxis or cooperate via attractant signal-based motion. Below, we investigate how these two mechanisms affect the biofilm’s morphology.

### Attractant signal: single and multiple stalks

During stalk growth, the attractant signal level in the centre of the stalk gradually increases. This induces an attractant signal gradient, which is sensed by the motile cells and attracts them radially towards the colony. The motile cells cover the stalk’s surface but show no preference for the top of the stalk (Fig. 2C) because of the uniform diffusion of the attractant signal through the stalk. The same morphology emerges in simulations with multiple stalks (or multiple initial inoculations). Hence the attraction of motile cells with a stalk-derived signal alone leads to aggregation of the motile cells in proximity to the stalks but fails to form the mushroom-shaped structures on the top of the stalks. In the following simulations, we switch off the attractant signal-based motion of cells. This assumption does not exclude the existence of such an attraction signal (e.g. quorum sensing). Instead, it means that an attraction signal alone is not sufficient to explain the formation of mushroom-shaped structures in *P*. *aeruginosa* biofilms.

### Nutrient chemotaxis: single stalks

**Figure 3:**
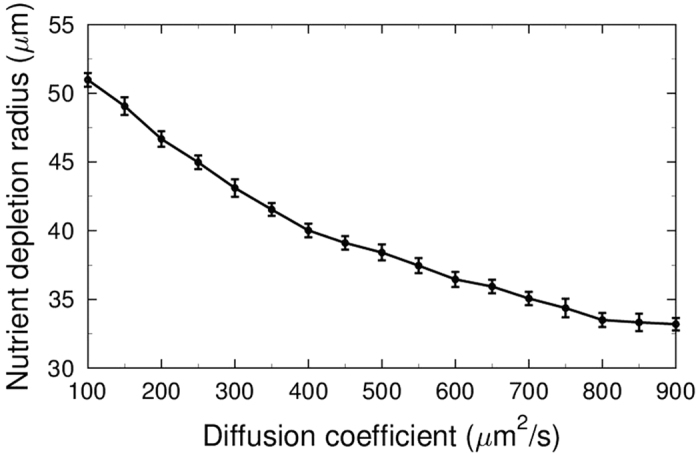
Diffusion-dependent radius of the nutrient-depleted region around a single stalk. A group of motile cells was positioned on the substratum, around a single stalk 50 μm in diameter. Cell growth and division were turned off to have a constant cell number. Simulations with *c*_*bulk*_ = 0.2 *mM* and different values of the nutrient diffusion coefficient (x-axis) were performed. The average radius of nutrient-depleted region (y-axis) was calculated over 10 replicates. Depletion is when the density of motile cells in a region drops below the 10% of their average density over the substratum.

In this alternative scenario, we assume that the motile cells only follow the nutrient gradient by nutrient chemotaxis. We perform one inoculation of each cell type (motile and immotile). During the early stages, while the immotile colony is forming a stalk, the motile cells move all over the substratum as well as the stalk’s surface. This continues until the cell number becomes so high that the nutrient level near the stalk drops below the chemotaxis threshold (*c*_*u*_; see Chemotaxis). The motile cells then chemotactically depart from the stalk. Because of its symmetric morphology, the stalk induces a ring around itself which motile cells evacuate from (Fig. 2D). The radius of this ring depends on the nutrient diffusion coefficient *D* (Fig. 3). The nutrient gradient around a single stalk, which radially repels the motile cell from it, cannot be equilibrated or inverted by further cell growth. The final configuration is a uniform layer of motile cells, with an evacuated region around the stalk. This shows that nutrient chemotaxis does not lead to mushroom formation in the case of single or sparse stalk inoculations.

### Nutrient chemotaxis: multiple stalks

**Figure 4:**
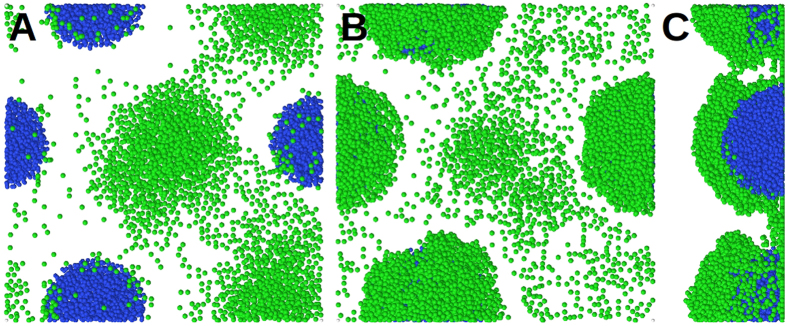
*In-silico* mushroom formation. (**A**) Initial growth and division of motile (green) and immotile (blue) cells after inoculation. The motile cells are repelled from stalks by nutrient depletion in the stalk’s vicinity. Motile cells move mainly in the inter-stalk area. (**B**) Because of further nutrient reduction at the bottom of the simulation box, immotile stalks stop growing and the motile cells migrate to the top of the stalks. (**C**) A side view of the biofilm in panel B reveals the cap formation on the stalks.

**Figure 5:**
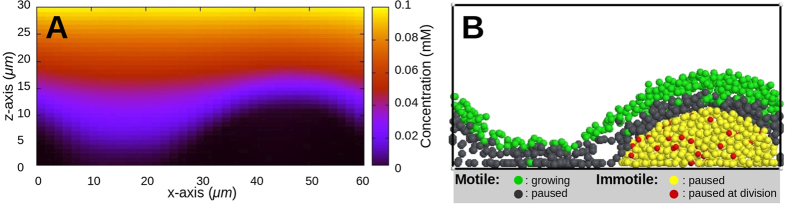
Nutrient concentration inside the stalks. (**A**) Nutrient concentration in the x-z plane through a stalk is shown, and coded by heat colours as shown at the right hand side. The inner parts of the stalk are nutrient deprived. (**B**) The nutrient deprivation inside the stalks puts the immotile cells into a resting phase and limits the growth of the stalks. Green and grey colours represent the motile cells with sufficient and deficient nutrient levels, respectively. Red and yellow colours represent the immotile cells in paused state, during and after division state, respectively.

Next, nutrient chemotaxis was investigated under denser stalk inoculations. Five initial inoculations were randomly positioned and grown on the substratum (number density: 3.5 × 10^−4^ *μm*^−2^; Fig. 4). Similar to the case of a single inoculation, the nutrient gradient initially repels motile cells from the stalks (Fig. 4A). However, nutrient-depleted regions of the neighbouring stalks overlap and the motile cells continue their chemotactic movement among the stalks. This leads to a collective motion of the motile cells towards the inter-stalk space on the substratum, without active migration onto the stalks. The biomass of motile and immotile cells grows continuously and the nutrient level near the substratum falls below the critical level (*c*_*l*_; see Chemotaxis). While the effect of the nutrient gradient vanishes in the horizontal (*xy)* direction, it increases in the *z*-direction (Fig. 5). This leads to an upward migration of the motile cells onto the stalks (Fig. 4B) and formation of mushroom caps (Fig. 4C). At the same time, the density of motile cells in the inter-stalk space decreases. The series of events mentioned above demonstrates how the behaviour of a cell subpopulation in a biofilm is influenced by the morphology of the biofilm itself.

### Quantification of mushroom structures

To investigate the effect of the number density of stalks on biofilm morphology more systematically, a measure for the degree of mushroom structures (*M*) is introduced. We define two independent quantities, *A* and *B*. *A* measures the fraction of substratum area covered by immotile cells; *B* measures the fraction of biomass of the motile cells that are located on the stalks. Both quantities are dimensionless and vary between 0 and 1. *M* is defined as the ratio *B*/*A*. With this definition, *M*~1 reflects an almost uniform distribution of the motile cells on the surface of the substratum and stalks. Values of *M* > 1 show a preference for stalk surfaces over the substratum for positioning of the motile cells, whereas *M* < 1 indicates the opposite.

**Figure 6:**
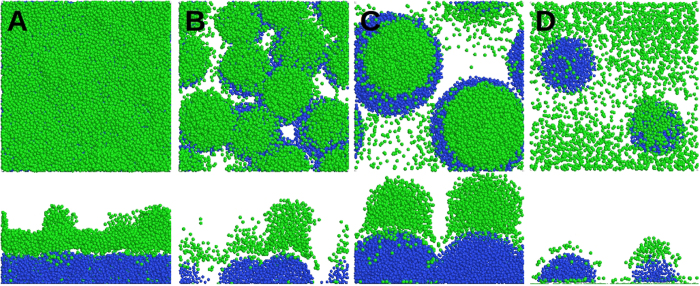
Quantification of mushroom shape with *M*-ratio. Biofilms formed by the reference system in Model Description, starting with (**A**) 20, (**B**) 10 and (**C**) 2 inoculations of immotile cells. They correspond to *M* values of 1.01, 1.13 and 1.5, respectively. (**D**) The same as in panel C, with a lower bulk nutrient concentration; *M* = 0.94. Blue and green colours represent the immotile and motile cells, respectively. x-z sections are shown at the bottom of the images.

In Fig. 6A, the motile cells are distributed over a layer of immotile cells, formed by an initial inoculation of 20 immotile cells. Because of their comparable sizes and high packing, the stalks form a uniform layer, on which the motile cells are distributed: *M* = 1.01. When we start the same simulation with 10 inoculations of the immotile cells (Fig. 6B), distinguishable stalks are formed, with the majority of the motile cells located on top of them, which leads to *M* = 1.13. When we decrease the number of initial inoculation points to two (Fig. 6C), clear mushroom structures are formed, where the motile cells preferentially segregate on their top: *M* = 1.5. If we critically reduce the bulk nutrient level in the latter simulation, the number of immotile and motile cells decreases as well (Fig. 6D). The small and sparse stalks formed in this case do not lead to a strong nutrient gradient in the *z*-direction and therefore, motile cells remain mainly on the substratum. Therefore, no clear caps are formed on the stalks, leading to *M* = 0.94. The proposed *M*-ratio is suitable for quantifying the formation of distinguishable stalks with caps of motile cells and a relatively evacuated substratum.

### Biofilm structure depends on inoculation density

**Figure 7:**
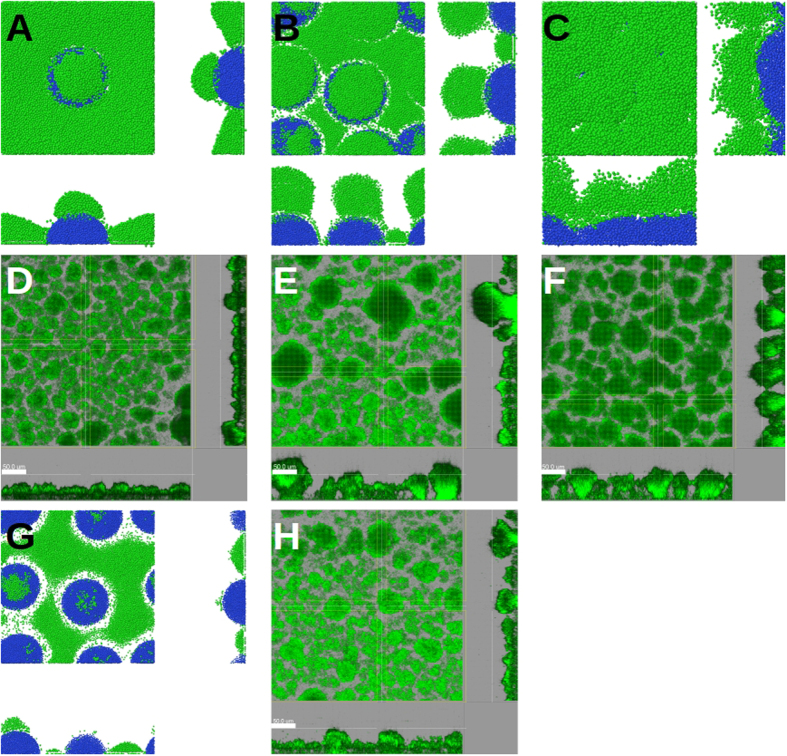
Biofilm fate influenced by host environmental factors. Snapshots of simulated biofilms formed by (**A**) low, (**B**) intermediate and (**C**) high (0.69, 2.8 and 6.9 × 10^−4^ *μm*^−2^, respectively) number density of initial inoculations, and bulk nutrient concentration of 0.2 mM. CLSM micrographs of 3-day-old *P. aeruginosa* biofilms, initiated with inocula with optical densities of (**D**) 0.0001, (**E**) 0.005 and (**F**) 0.05. (**G**) A snapshot of the same simulated biofilm as in panel B, but under low (0.1 mM) bulk nutrient concentrations. (**H**) CLSM micrograph of the same 3-day-old *P. aeruginosa* biofilms as in panel E, but under low nutrient conditions. In A, B, C and G blue and green colours represent the immotile and motile cells, respectively. In D, E, F and H the biofilms are visualized via a constitutively expressed GFP. The size of the simulation box is 120 μm. The bars represent 50 μm. The central images show the top (x-y) view; x-z and y-z sections are shown at the bottom and right-hand side of the images.

Next, we repeated the simulation using our reference model, with three different inoculation densities. At very low densities (Fig. 7A; 0.69 × 10^−4^ *μm*^−2^), the stalks are so distant that their nutrient-depleted regions do not overlap. The majority of the motile cells stay in nutrient-rich regions away from the stalks. The corresponding *M*-ratio at the end of the simulation is *M* = 0.65. At a very high initial number density of inoculations (Fig. 7C; 6.9 × 10^−4^ *μm*^−2^), the stalks overlap very quickly. This leads to mostly connected and small immotile colonies forming a rough layer, covered with motile cells. In this case, *M* = 1.02 is achieved at the end of simulation. Hence, in both extreme values of stalk density, mushroom structures rarely form. However, with an intermediate initial number density of inoculations (Fig. 7B; 2.8 × 10^−4^ *μm*^−2^), clear and distinguishable mushroom structures (*M* = 1.3) are formed. Therefore, the formation of mushroom-shaped structures depends on the number density of stalks.

The predicted dependence of biofilm structure on the inoculation density is confirmed by CLSM micrographs of 3-day-old *P*. *aeruginosa* biofilms, as shown in Fig. 7 (panels D–F). When we initiated biofilm growth with different inoculum optical densities, biofilms of different morphologies were observed. At very low inoculum optical densities (Fig. 7D), a uniform distribution of tiny microcolonies formed on the substratum. Increasing the inoculum optical density led to the emergence of bigger mushroom-shaped structures, surrounded by small and sparse microcolonies on the substratum (Fig. 7E). A further increase of the inoculum optical density resulted in more uniform and tightly connected mushroom-shaped structures (Fig. 7F). In agreement with our model and experimental observations, multiple (well-spaced and non-overlapping) mushroom structures have always been visible in previously published CLSM images of real biofilms^13^,^33^, rather than single or isolated ones. Please refer to the *Supplementary Information* for further details of quantifying the morphological differences between panels D, E, F and H of Fig. 7.

### Bulk nutrient concentration

As the number density of stalks affects the biofilm morphology via the nutrient-depleted regions around the stalks, we hypothesised that the nutrient concentration itself would also influence the ultimate morphology of the biofilm. Starting from the simulation in Fig. 7B (intermediate number density of initial inoculations) the bulk nutrient level was reduced to 0.1 mM. Stalks grew more slowly than before. The nutrient concentration fell below the sensitivity threshold for motile cells such that they could not follow the gradient. This led to a random motion of motile cells and their quasi-uniform distribution on the substratum and the stalks (Fig. 7G). The resulting *M*-ratio was *M* = 0.17. Micrographs of CLSM confirms this bulk nutrient-dependence of the biofilm’s fate: While the biofilm next to the inlet receives the fresh medium with high nutrient levels (Fig. 7E), the biofilm further downstream receives less due to nutrient consumption, leading to more tiny and connected colonies (Fig. 7H).

### Interplay of inoculation density and nutrients

**Figure 8:**
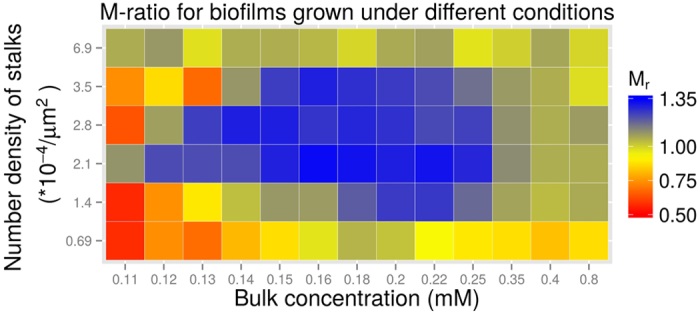
Biofilm morphology influenced by the number density of the stalks (*σ*_*n*_), and bulk nutrient level. The morphology of the mushroom structures is quantified as explained in Quantification of mushroom structures.

As the model generates results that are consistent with experimental findings, we extended the analysis of bulk nutrient levels *c*_*bulk*_ and inoculation densities *σ*_*n*_ to predict a complete picture of how mushroom formation depends on these two factors. The *M*-ratios were measured and are summarised in Fig. 8. It was found that formation of mushroom-shaped structures is not only limited to a range of the number densities of stalks, but also to a range of bulk nutrient levels. At intermediate number densities of stalks, mushrooms seldom form for low nutrients and are reduced again for very high nutrient levels. Thus an optimal regime of these two parameters for the formation of mushroom-shaped structures was identified.

## Summary and Outlook

An agent-based model for biofilm growth of *P*. *aeruginosa* bacteria was presented that includes motile and immotile cells, where the motile cells have the possibility of moving on the substratum and on top of the immotile cell colonies. As driving forces for active motility, competitive nutrient chemotaxis and cooperative attractant signal mechanisms were considered. Mushroom-shaped structures formed only in the presence of both cell types: motile and immotile. In contrast to previous models^24^, we were able to find mushroom-shaped biofilm structures in response to nutrient chemotaxis at a range of stalk number densities. The fact that attractant signal failed to generate such structures supports the hypothesis that competition among *P*. *aeruginosa* bacteria is more important for mushroom formation than cooperativity.

Formation of mushroom-shaped structures was found to depend on the number density of stalks and on the nutrient level. This result explains the seemingly contradictory observations of the collective motion of *P*. *aeruginosa* cells onto the stalks and nutrient gradients away from the stalks^13^,^35^,^36^. The latter corresponds to a configuration at low stalk density, where nutrient levels among the stalks are sufficiently high. The observed collective motion onto the stalks corresponds to a configuration at high stalk density, where the nutrient level among the stalks is widely depleted and forces motile cells to migrate into the third dimension in order to detect more favourable nutrient conditions. The cap-forming motile cells have better access to nutrient, and their biomass may increase, depending on the bulk nutrient level.

Outside the optimal regime of stalk density and nutrient level, mushroom formation is prevented by random migration of the motile cells, which tend to cover the area irrespective of the presence of stalks. In that sense, the stalks are still covered by motile cells but the morphology does not correspond to a classical mushroom structure. This is well captured by the *M*-ratio introduced for the purpose of quantifying the degree of formation of mushroom-shaped biofilm structures.

The mushroom-shaped structures form under hydrodynamic conditions in flow-cell systems. In earlier studies, it has been reported that such mushroom-shaped structures form only when glucose is used as a carbon source. For example, Klausen *et al*.^33^ observed that when PAO1 cells are grown in a citrate minimal medium, formation of mushrooms is prevented by bacterial migration: it leads to a uniform and flat biofilm covering the substratum. However, in our laboratory setup, we never observed flat biofilms when we used citrate as a carbon source.

Our results are in agreement with previously published experimental observations that a motile bacterial subpopulation forms caps on top of stalks formed by an immotile bacterial subpopulation^13^,^33^. The role of the chemotaxis system in the development of mushroom-shaped structures in *P*. *aeruginosa* biofilms was shown in *cheY* mutants^35^. These mutants were deficient in chemotaxis but were capable of swimming and surface motility. *P*. *aeruginosa cheY* mutants were unable to form caps on the top of stalks formed by the same mutants or by *pilA* (lacking Type IV pili) mutants^35^. This corresponds to the cases in our model where we turn off the chemotaxis systems of cells (data not shown) or set extreme levels of bulk nutrient such that cells no longer sense a nutrient gradient (Figs 7 and 8). In both of these cases, biofilm morphology deviated from clear mushroom shapes and had low M-ratios.

Other environmental factors might be added in future work to the landscape of mushroom-shaped biofilm formation. Such additional factors may include the adhesion of bacteria to the substrate or each other, which were set to be constant in the present investigation. It was shown that extracellular DNA plays a role in the initialisation of immotile cell colonies^14^, as well as the absorption of motile cells to the surface of such colonies^48^. Consideration of the variation in such factors would further improve the predictive power of the model. Indeed, biofilm morphology has a significant impact on biofilm behaviour and evolution^28^,^50^,^51^, as well as antibiotic resistance^4^,^9^,^10^,^11^. In this context, new treatment methods exploiting the social behaviour of biofilms or their interactions with the hosting medium are appealing^26^,^52^,^53^.

Our model investigates how diffusion-induced gradients affect the collective migration of bacterial cells and shape the biofilm’s morphology under different conditions. Cells are assumed to keep their motility state throughout simulations, because little is known about their decision making mechanisms. A potential application of our model would be testing different scenarios of trail-mediated mutual and self-interaction of the cells and their phenotypic switch from the motile to the immotile state. The latter is the first step in colonising a surface, leading to *stalks*, and is not well understood^14^. In the current model, the number density of stalks can be arbitrarily large. In reality, the number of these stalks, which is determined by the fraction of cells entering an immotile state, might saturate beyond a given inoculation density. An *in silico* mechanism by which cells determine their motility state will lead to a dynamic and probably reversible contribution in the biofilm morphology and maturation. A key player bridging the transition from a motile planktonic lifestyle to a sessile one is the second messenger molecule cyclic di-GMP (c-di-GMP). The intracellular level of c-di-GMP is controlled via various enzymes synthesising (diguanylate cyclases) and degrading (phosphodiesterases) the molecule, thereby affecting various cellular processes in response to environmental and cellular changes^54^,^55^. There is evidence that the extracellular polysaccharide produced by neighbouring cells can increase the intracellular c-di-GMP level of *P*. *aeruginosa* cells^56^, which extends the cell state dynamics to cell–cell interactions. The combination of model prediction and experimental data might help to understand the decision making mechanisms in this very first step of biofilm formation. An agent-based model is suitable for this purpose because of recent single-cell level experiments on microcolony formation. Another recent study showed that formation of spatial patterns and cooperation in surface-attached colonies of *Bacillus subtilis* is affected by the initial founder cell densities^51^. The lack of motile cells in that study corresponds to cases that were subsequent to surface colonisation. Another potential application of our model would be to investigate how the spatial organisation of cooperative and non-cooperative cells is affected by cell motility before or during surface colonisation.

A reliable prediction with a detailed model considering both internal and external agents can minimise experimental testing and thus strongly speed up biofilm research. By now, the experimental workflow of flow cell experiments is still very time-consuming due to low sample numbers and a complex setup, and remains the bottle-neck in testing various settings at a time. Changing the given agents might also enable the adaptation of the present model to other *in vitro* biofilm systems, since there are many more models available^57^. Moreover, there is rising evidence that multispecies biofilms are growing in importance, corrupting possible treatments^58^. Further development to predict the coevolution/coexistence of two species or more differing in shape and growth rate, and expressing attractive, competitive or even destructive agents is of high importance.

Our findings in this study connect the fate of biofilms to the initial conditions under which they start to grow. We have used the initial number of stalks as an initial condition in our simulations. In reality, the number and size of the initial microcolonies in *P. aeruginosa* biofilms occupying a given surface correlate with communally produced Psl-rich regions^14^. This opens the possibility of predicting and possibly manipulating the emergence of mushroom-shaped structures, depending on the environment. A considerable advantage of manipulating biofilm morphology via nutrient levels and stalk density, as proposed in this work, is that these non-microbicidal mechanisms do not impose evolutionary pressure on the bacteria and therefore do not induce antibiotic resistance^59^.

## Additional Information

**How to cite this article**: Ghanbari, A. *et al*. Inoculation density and nutrient level determine the formation of mushroom-shaped structures in *Pseudomonas aeruginosa* biofilms. *Sci. Rep.* **6**, 32097; doi: 10.1038/srep32097 (2016).

## Supplementary Material

Supplementary Information
