# Supplementary material for: Inoculation density and nutrient level determine the formation of mushroom-shaped structures in Pseudomonas aeruginosa biofilms
Source: Sci Rep. 2016 Sep 9;6:32097. doi: 10.1038/srep32097 (PMC5017200; doi:10.1038/srep32097)
Supplement: Supplementary Information [file srep32097-s1.pdf]

Supplementary information

Inoculation density and nutrient level determine the formation of mushroom-shaped structures in *Pseudomonas aeruginosa* biofilms

Azadeh Ghanbari[1,*], Jaber Dehghany[1,*], Timo Schwebs[2], Mathias Müsken[2,3], Susanne Häussler[2,3] and Michael Meyer-Hermann[1,4]

[1)] Department of Systems Immunology and Braunschweig Integrated Centre of Systems Biology, Helmholtz Centre for Infection Research, Braunschweig, Germany

[2)] Institute for Molecular Bacteriology, Twincore, Centre for Experimental and Clinical Infection Research, Hannover, Germany

[3)] Department of Molecular Bacteriology, Helmholtz Centre for Infection Research, Braunschweig, Germany

[4)] Institute for Biochemistry, Biotechnology and Bioinformatics, Technische Universität Braunschweig, Braunschweig, Germany

* These authors contributed equally to this work.

Table S1: Model parameters.

Symbol	Description	Value	Units	Reference
dt	Time step	variable	s	-
l_x, l_y, l_z	Simulation box size	150, 150, 200	µm	-
R_0	Cell radius	0.68	µm	approximated
l_d	Diffusion lattice size	2	µm	-
h	Superficial mesh size	$R_0/2$	µm	-
d_c	Cut-off distance	$3\times R_0$	µm	-
d_{max}	Maximum displacement per time step	R_0	µm	-
v	Cell speed	0.04	µm/s	Conrad 2011
l_p	Persistence length	4	µm	Conrad 2011
D	Diffusion coefficient	678	$\mu m^2/s$	Kreft 1998
α	Relative diffusivity factor	0.25	-	Stewart 2003
τ_d	Cell division time	30	min	assumed
c_b	Bulk nutrient level	variable	mM	assumed
c_u	Nutrient rich, threshold	$0.5\times c_b$	mM	assumed
c_l	Nutrient depletion, threshold	0.05	mM	assumed
Y_{max}	Growth yield	0.444	-	Kreft 1998
V_{max}	Maximum substrate uptake rate	0.00077	s^{-1}	Kreft 1998
K_s	Half-saturation constant	0.00234	fg/fl	Kreft 1998
m	Cell maintenance rate	0.00001	s^{-1}	Kreft 1998
ρ	Cell mass density	290	fg/fl	Kreft 1998

The persistence time was calculated as l_p/v.

Quantification of morphological characteristics of *P. aeruginosa* biofilms

3-day-old PA biofilms, initiated with inocula with optical densities of (D) 0.0001, (E) 0.005 and (F) 0.05, grown under high nutrient levels, as well as with an optical density of (H) 0.005 but grown under low nutrient conditions, were analysed. The low nutrient conditions were ensured by acquiring images at the very end of the flow cell, where nutrient levels were reduced due to consumption at earlier stages. The abbreviations D, E, F and H were chosen to be consistent with the panels of Fig. 7 in the main text. Analysis was performed by Imaris software, where the same settings were applied for all image stacks to determine the surfaces and calculate the objects' volumes (surface grain size = 0.757 um; manual threshold: value for the GFP channel = 15 (absolute intensity); splitting: estimated diameter of the region growing = 25.0 um; classification: "number of voxels" above 10,000).

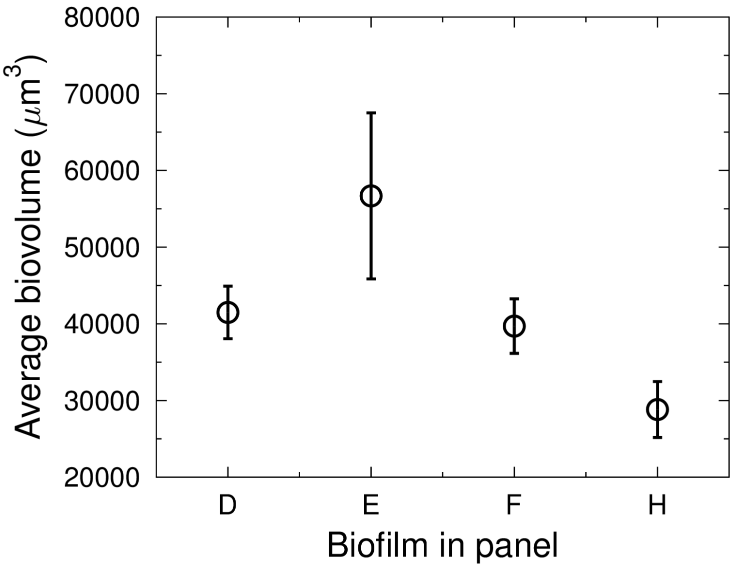

Figure S1: *Average biovolume of objects (microcolonies) in Experiment 1 after filtering very small (<10,000 μm^3) objects. The x-axis refers to the different panels of Fig. 7 in the main text. There were 54, 37, 66 and 43 microcolonies in Panels D, E, F and H, respectively.*

Our analysis shows that the number of events, after removing very small ones (<10,000 μm^3), reaches a minimum in the presence of an intermediate nutrient level (Fig. 7; 37 objects in Panel E, compared to 54 and 66 in Panels D and F, respectively). At the same time, the average volume of

these structures peaks at intermediate nutrient levels (Fig. 7; 56,681 μm^3 in Panel E, compared to 41,486 and 39701 μm^3 in Panels D and F, respectively). Fig. S1 shows the average biovolume of the microcolonies in Fig. 7-D, E, F and H of the main text.

Fig. S2 shows the distribution of object volumes (from Fig. 7-D, E, F and H of the main text) after filtering very small (<10,000 μm^3) objects. The first four very large structures belong to Panel E.

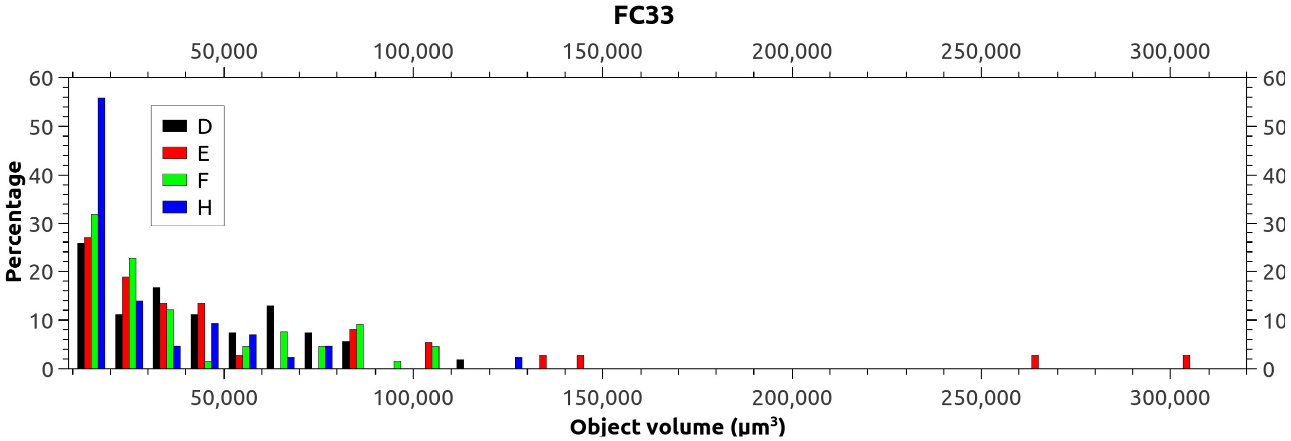

Figure S2: *Distribution of object volumes in Experiment 1 after filtering very small (<10,000 μm^3) objects. The bar colours refer to the different panels of Fig. 7 in the main text (see inset).*

We have performed a replicate experiment and, consistent with the first experiment, very large objects were observed only in the case of Panel E (Fig. S3).

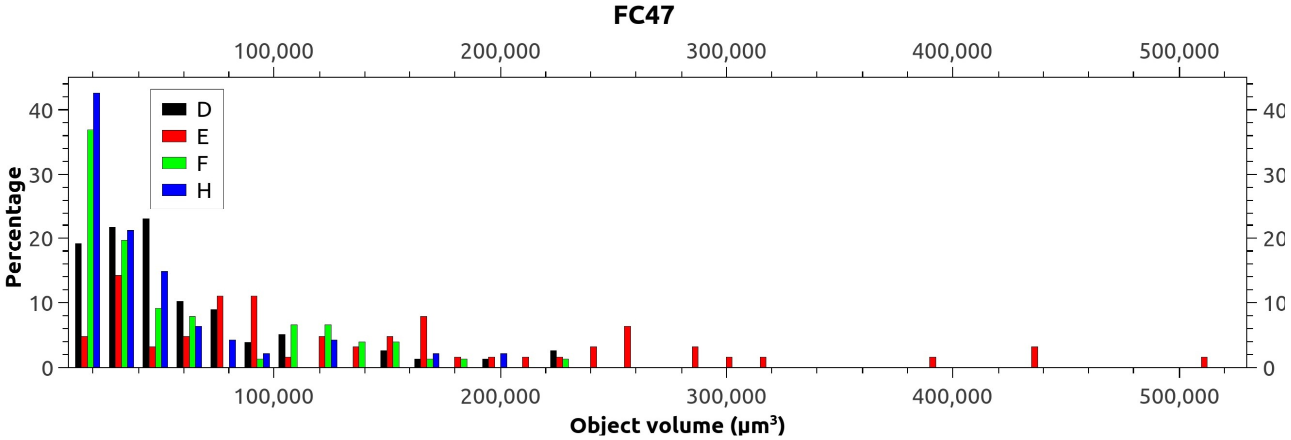

Figure S3: *Distribution of object volumes in Experiment 2 after filtering very small (<10,000 μm^3) objects. Bar colours as in Fig. S2.*

Fig. S4 compares the volumes of the top 10% of the largest objects of each Panel (D, E, F and H) for merged datasets. It shows that while the largest objects in Panels D, F and H are comparable in size, those in Panel E are significantly larger.

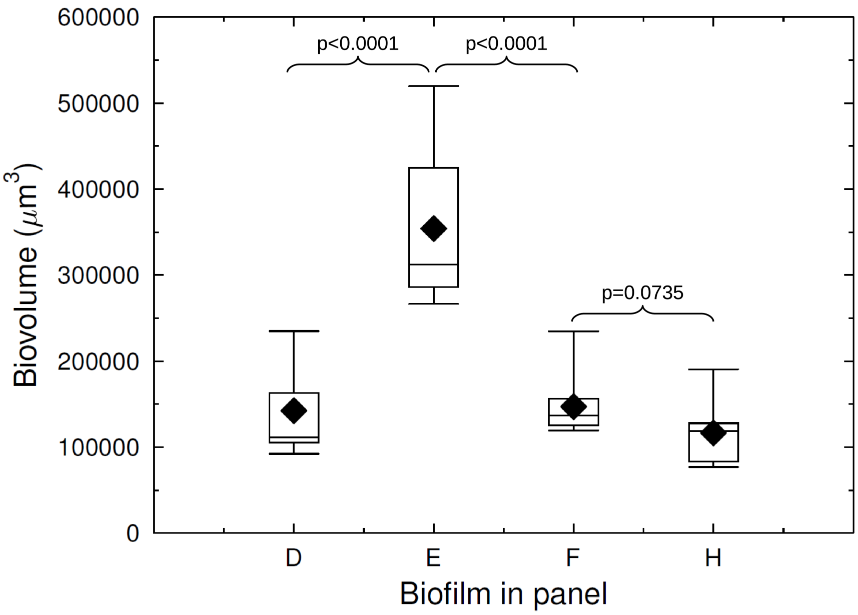

Figure S4: *Biovolume of the top 10% of the largest objects in Panels D, E, F and H of Fig. 7 of the main text.*
